# Cell-Specific mRNA Profiling of the *Caenorhabditis elegans* Somatic Gonadal Precursor Cells Identifies Suites of Sex-Biased and Gonad-Enriched Transcripts

**DOI:** 10.1534/g3.115.022517

**Published:** 2015-10-23

**Authors:** Mary B. Kroetz, David Zarkower

**Affiliations:** * Department of Genetics, Cell Biology and Development, University of Minnesota, Minneapolis, Minnesota 55455; † Masonic Cancer Center, University of Minnesota, Minneapolis, Minnesota 55455

**Keywords:** sex determination, gonad, RNA-seq, Z1/Z4, *C. elegans*, genetics of sex

## Abstract

The *Caenorhabditis elegans* somatic gonad differs greatly between the two sexes in its pattern of cell divisions, migration, and differentiation. Despite decades of study, the genetic pathways directing early gonadal development and establishing sexual dimorphism in the gonad remain largely unknown. To help define the genetic networks that regulate gonadal development, we employed cell-specific RNA-seq. We identified transcripts present in the somatic gonadal precursor cells and their daughter cells of each sex at the onset of sexual differentiation. We identified several hundred gonad-enriched transcripts, including the majority of known regulators of early gonadal development, and transgenic reporter analysis confirmed the effectiveness of this approach. Before the division of the somatic gonad precursors, few sex-biased gonadal transcripts were detectable; less than 6 hr later, after their division, we identified more than 250 sex-biased transcripts, of which about a third were enriched in the somatic gonad compared to the whole animal. This indicates that a robust sex-biased developmental program, some of it gonad-specific, initiates in the somatic gonadal precursor cells around the time of their first division. About 10% of male-biased transcripts had orthologs with male-biased expression in the early mouse gonad, suggesting possible conservation of gonad sex differentiation. Cell-specific analysis also identified approximately 70 previously unannotated mRNA isoforms that are enriched in the somatic gonad. Our data illustrate the power of cell-specific transcriptome analysis and suggest that early sex differentiation in the gonad is controlled by a relatively small suite of differentially expressed genes, even after dimorphism has become apparent.

The *Caenorhabditis elegans* gonad originates during embryogenesis as a four-celled structure composed of two somatic gonadal precursor cells (Z1 and Z4) flanking two germline precursor cells (Z2 and Z3). The four-celled gonadal primordium is morphologically identical between males and hermaphrodites. However genetic analysis indicates that gonadal sex is determined during a short interval centered around hatching, a time when the gonad still appears sexually indistinct (Klass *et al.* 1976; Nelson *et al.* 1978). After hatching, the gonadal precursor cells are then poised to develop into one of two sex-specific organ structures: paired ovotestes in the hermaphrodite or a single testis in the male. Gonadogenesis involves major sex differences in the pattern of cell divisions, cell migration and the differentiated cell types that are formed (Kimble and Hirsh 1979). Despite much study, the genetic pathways that direct early gonadal development and establish sexual dimorphism in the gonad remain largely unknown, with just a handful of regulatory genes identified so far from genetic screens (reviewed by Emmons 2014).

Cell-specific RNA-seq is a technique that has been pioneered for neuronal transcriptomes and a number of other cell types in *C. elegans* (Spencer *et al.* 2011, 2014). Here we use RNA-seq of purified cells to define the transcriptome of the somatic gonad primordium in each sex in order to delineate components of the distinct genetic networks that regulate organ-specific and sex-specific gonadal development. We examined two key time points in early larval development: before and after the first division of Z1 and Z4. We hypothesized that at the earlier time we would identify initial regulators of gonadogenesis, and at the later time, which is after the gonad has become morphologically distinct between the sexes, we would identify regulators and effectors that continue to promote sexual dimorphism. Our RNA-seq analysis identified transcripts enriched in the gonad compared to the whole animal, including the majority of the known regulators of early gonadal differentiation. We also identified transcripts with differential expression between the sexes in the gonad, which will be referred to as sex-biased expression. TRA-1 is a transcription factor that determines sex throughout the body, including in the gonad (Hodgkin 1987; Zarkower and Hodgkin 1992). Surprisingly, very few transcripts enriched in the somatic gonad had sex-biased expression at the earlier time point, suggesting that TRA-1 may be regulating only a small subset of genes within the gonad. Perhaps the initial events in dimorphic gonadogenesis may largely involve other modes of gene regulation. However, after the division of Z1/Z4 we observed a 10-fold increase in the number of sex-biased transcripts. We found that about 10% of male-biased transcripts have mammalian counterparts with male-biased expression in the analogous cells of the fetal mouse gonad. The vast majority of the sex-biased expression differences we detected within the gonad could not be detected in the intact animal, highlighting the importance of developing techniques to isolate and profile distinct cell populations. In this work, optimizing and implementing a new isolation protocol for individual larval gonadal cells has allowed us to transcriptionally profile an organ primordium and determine the sex-biased profile of a somatic tissue in *C. elegans* for the first time.

## Materials and Methods

### Strains

Z1/Z4 and their daughter cells were isolated for transcriptional profiling from *DZ685 [xol-1(y9) X***;** *rdIs4 (ehn-3a(promoter*::*Venus)]* in hermaphrodites. The *rdIs4* expresses the Venus fluorescent protein exclusively in Z1/Z4 and their descendants. To collect large populations of phenotypically male animals DZ683 {*tra-2(ar221) II*; *xol-1(y9) X*; *rdIs4* [*ehn-3a(promoter)*::*Venus*]} was used. This strain has a temperature-sensitive mutation in *tra-2*, a component of the global sex determination pathway. *tra-2(ar221) xol-1(y9)* animals reared at permissive temperature develop as hermaphrodites, and those reared at restrictive temperature develop as well masculinized XX pseudomales (Hodgkin 2002), which will be referred to as males. The *xol-1(y9)* mutation kills spontaneous XO males by disrupting sex chromosome dosage compensation and also enhances masculinization by *tra-2(ar221)*. Dissociated cells from DZ685 and DZ683 that were not sorted by fluorescence-activated cell sorting (FACS) were used to collect whole animal populations of cells for the later time point, and both time points, respectively. *xol-1(y9)X*, a strain lacking *rdIs4*, was used for the hermaphrodite whole animal sample for the earlier time point.

### Nematode culture

To produce large populations of animals for isolation of cells from the gonadal primordium, cultures of synchronized animals were reared by the following method: animals were first cultured on standard 60 mm nematode growth medium (NGM) plates (Stiernagle 2006) until food was exhausted. Each starved 60-mm NGM plate was used to inoculate two 150-mm bactopeptone-rich 8P plates with four times the usual amount of cholesterol supplementation (Bianchi and Driscoll 2006). Strains were grown at 15° until plates were full of gravid adults (about 5 days), shifted to room temperature for about 90 min and then animals were washed from the plates with M9 (Stiernagle 2006). To obtain sufficient material for transcriptional profiling, approximately 20–40 million embryos of each sex were collected for each sample replicate. Eggs were isolated by bleaching, washed repeatedly with M9 to remove any trace of bleach, (Stiernagle 2006) and allowed to hatch in ∼300 ml M9 overnight while shaking at 250 rpm at the restrictive temperature of 23.5°. Since RNA was isolated directly from FACS-sorted dissociated cells without cell culture, hatched larvae could be fed a normal bacterial diet instead of an axenic medium. Hatched larvae were plated on NA22-seeded 150-mm 8P plates in small droplets with approximately 1–4 million larvae per plate and incubated at 23.5° for 9.5 or 15 hr prior to isolation of Z1/Z4 or their daughters.

### L1 cell isolation

Mid- and late-L1 animals fed for either 9.5 or 15 hr were recovered from plates with ice-cold M9, washed three to four times with ice-cold M9, and transferred to a chilled 15 ml conical tube. Live larvae were then collected by sucrose floatation in 9 ml of ice cold 70% sucrose mixed with 6 ml of ice-cold M9 and centrifuged immediately at 4° for 5 min at 2000 rpm. Up to 10 ml of solution containing larvae was removed from the tube with polished blunt Pasteur pipet. Collected larvae were diluted to 50 ml with M9 in a 50-ml conical tube and washed twice for 2 min at 2000 rpm to remove sucrose. Approximately 1.2 ml of packed larvae were pelleted by centrifugation at 7000 rpm for 1 min in 1.5 ml microfuge tubes, washed with sterile water and pelleted again, with a maximum volume of 200 µl of packed worms per tube.

Cell isolation from the collected larvae largely followed Zhang *et al.* (2011) with modifications to allow isolation of larger numbers of cells, which included adjusting volumes, incubations, amount of mechanical force by pipetting, and filtering of debris after dissociation. With these modifications, we were able to dissociate a five to ten times larger volume of cells per microfuge tube than what was previously published, which greatly aided in procuring enough mRNA for isolation and transcriptional profiling of a specific subset of cells which were less than 1% of the cells in the whole animal at this time. We also fed larvae a normal bacterial diet rather than the axenic medium used in previous isolations.

To disrupt the cuticle, each tube of washed and pelleted animals was incubated for 2 min in 500 µl of freshly thawed sterile SDS-DTT solution (Zhang *et al.* 2011), and then 1 ml of egg buffer (Strange *et al.* 2007) was added and tubes were spun immediately for 1 min at 7000 rpm. Supernatant was removed and pelleted animals were washed five times with 1 ml egg buffer. Pelleted SDS-DTT-treated worms were then suspended in 500 µl of 15 mg/ml pronase (P8811; Sigma-Aldrich, St. Louis, MO) in egg buffer and incubated 7 min at room temperature and then pipetted with a standard 1-ml plastic pipet for 15 min continuously in pronase solution to dissociate cells by mechanical force. Animals were incubated in pronase for an additional 3–15 min after pipetting. One milliliter of L-15 medium supplemented with 10% fetal bovine serum, 50 U/ml penicillin, and 50 µg/ml streptomycin (Strange *et al.* 2007) was added to each tube to quench pronase digestion, and cells were pelleted by centrifugation at 10,000 rpm for 5 min at 4°. Pellets were washed twice with L-15/FBS and resuspended in 1 ml L-15/FBS. A small sample of cell suspension was examined on a microscope slide to verify the presence of green fluorescent protein (GFP)-positive single cells and the overall effectiveness of the cell dissociation. Resuspended cells were filtered with a 5 µm filter (EMD Millipore) to separate individual cells from carcasses and debris, and filtered cells were pelleted by centrifugation at 10,000 rpm for 5 min at 4°. Cells were resuspended in egg buffer with 5 µg/ml of propidium iodide to identify and remove dead cells during cell sorting and then sorted by FACS at the University of Minnesota Flow Cytometry Resource on an AriaII cell sorter with a 70 µm diameter nozzle. To exclude auto-fluorescent cells during FACS, strains harboring *rdIs4* were compared to *xol-1(y9)X*, a strain lacking GFP.

### RNA extraction and sequencing

Sorted cells were collected directly into TRIzol (Ambion, Carlsbad, CA) for RNA extraction. RNA was purified using RNeasy MinElute spin column kits (Qiagen, Valencia, CA). rRNA was depleted by Ribo-Zero (Epicentre, Madison, WI). Initial experiments used Ovation RNA-Seq System V2 (NuGEN, San Carlos, CA) to amplify cDNA, but subsequent experiments proceeded directly to TruSeq (Illumina, San Diego, CA) from the rRNA-depleted sample but omitting the poly-A selection of mRNA. Next generation sequencing was performed at the University of Minnesota Genomics Center using single-end 50 bp reads. Sequences were mapped to the *C. elegans* genome (USCS ce10, Wormbase WS220) using Illumina-supplied annotations. Reads were mapped using TopHat v1.4.1 and differential expression was analyzed using Cuffdiff v2.1.1 (Trapnell *et al.* 2012).

### Gene ontology

To determine transcripts that were overrepresented in the gonad-enriched datasets, the PANTHER Overrepresentation Test (release 20150430) was used (Mi *et al.* 2013). The annotation version and release date were GO Ontology database released 2015-07-07. To determine transcripts that had sex-biased function, DAVID Bioinformatics Resources 6.7 was used (Huang da *et al.* 2009).

### Expression analysis and microscopy

Promoter regions containing between 400 bp and 3.6 kb of DNA from upstream of the start codon (Supporting Information, Table S1) were cloned into the GFP expression vector pPD95.75. Reporter constructs (20 ng/µl) were injected into the distal gonads of adult *him-8(e1489)* hermaphrodites and stable transgenic lines were assayed for *GFP* expression. Differential interference contrast (DIC) and fluorescent images were acquired on a Zeiss motorized Axioplan 2 microscope using an AxioCam MRm camera and AxioVision software (Zeiss, Jena, Germany).

### Motif analyses

DREME was used to identify over-represented motifs and then Tomtom was used to compare each DREME-generated motif against a database of known motifs. FIMO was then used to identify which promoter regions of transcripts contained the DREME-generated motif. All informatics tools can be found at: http://meme-suite.org.

### RNAi analyses

Bacterial clones (Kamath and Ahringer 2003) obtained from SourceBioSciences were grown overnight in Luria-Bertani (LB) medium and seeded onto NGM medium containing 0.02% lactose to induce dsRNA expression. Larval L4 hermaphrodite animals were placed on RNAi plates and incubated at 22°. On the following day, adult animals were moved to a second RNAi plate and rearing continued at 22°. Male young adult progeny were scored for phenotypic defects.

### Hyper-geometric calculations

To determine whether there is significant overlap between two datasets the following website was used: http://nemates.org/MA/progs/overlap_stats.html. A total of 19,862 genes per genome was used for all calculations. This was the total number of genes mapped in WS220 available in iGenomes for TopHat v1.4.1, which was used in all bioinformatics analyses. Comparisons were made to determine significant overlap between sex-biased transcripts and genes near TRA-1 binding sites (*P* < 0.05) as well as to determine whether sex-biased or gonad-enriched transcripts were significantly over-represented on a chromosome. (*P* values of < 0.01 and 0.001 are indicated by * and **, respectively).

### Data availability

Strains are available upon request. Illumina sequence data for RNA-seq have been deposited at NCBI with accession number GSE71772.

## Results

### Sexually dimorphic mRNA expression in the somatic gonadal primordium

**Figure 1:**
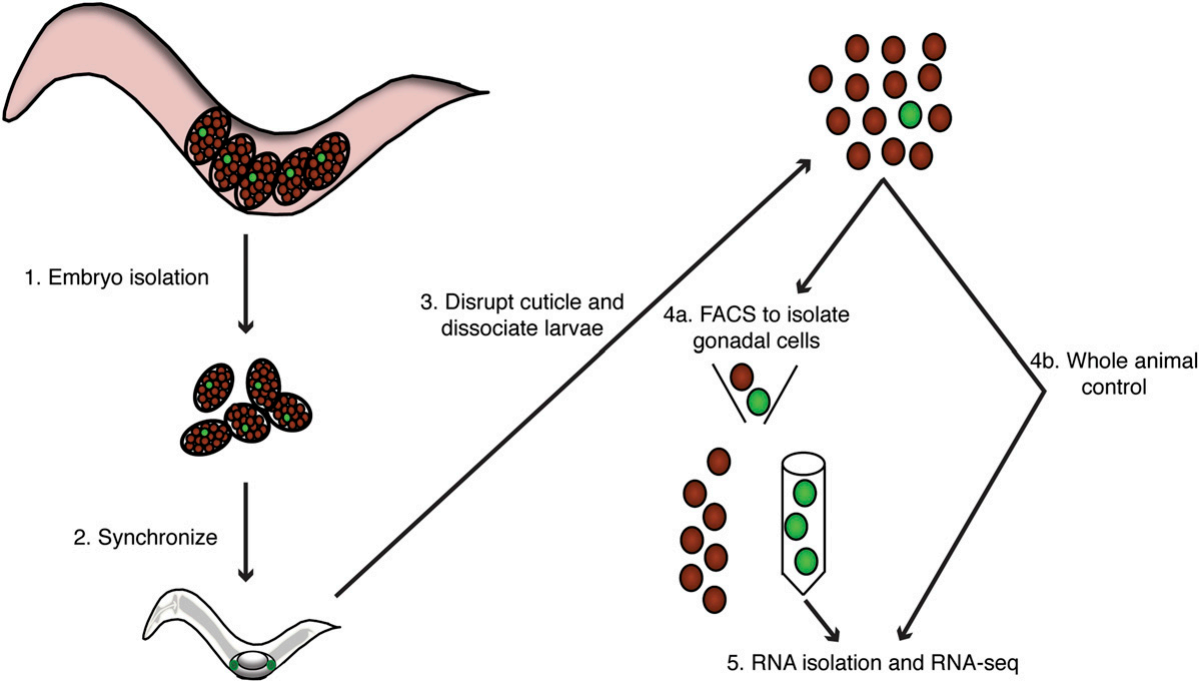
Schematic of cell-specific RNA-sequation. (A) Embryos harvested using strains that give rise to pure populations of either sex. (B) Animals were hatched in the absence of food to synchronize growth. After hatching, larvae were fed to allow development to proceed. (C) Animals were harvested at the appropriate developmental time point by disrupting the cuticle and dissociating and filtering the single cells. (D) The majority of the dissociated cell suspension was sorted by FACS to isolate the gonadal cells. An aliquot of the dissociated cells was removed prior to FACS to be used as a whole animal control. (E) RNA was isolated and RNA-seq performed (see *Materials and Methods*).

**Figure 2:**
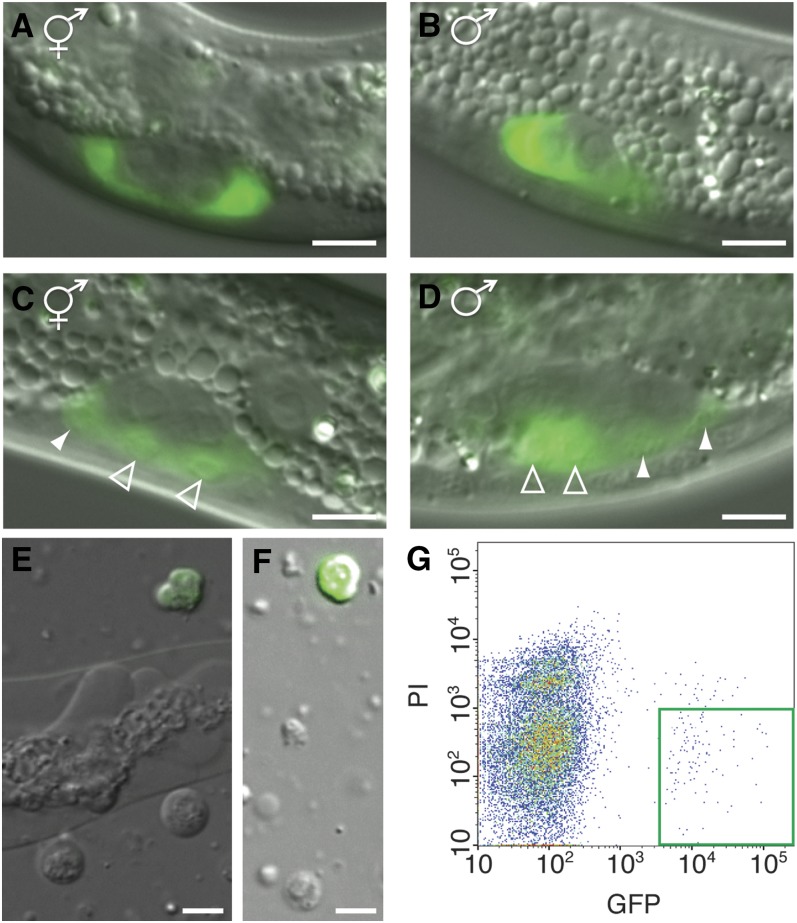
Green fluorescent protein (GFP) expression in somatic gonadal precursor cells. Differential interference contrast (DIC) and fluorescence micrographs of (A) gonad of a hermaphrodite larva at 9.5 hr postfeeding. (B) Gonad of a male larva at 9.5 hr postfeeding. Only Z1 is visible in the male, because Z4 lies outside focal plane. Prior to the division of Z1/Z4 the gonad appears identical between the sexes. (C) Gonad of a hermaphrodite larva at 15 hr postfeeding after the division of Z1/Z4. Distal daughters are marked by filled arrowheads and proximal daughters are marked by open arrowheads. (D) Gonad of a male larva at 15 hr postfeeding after Z1/Z4 daughters have migrated and gonads become sexually dimorphic. Note that distal daughters of Z1/Z4 are smaller. (E) Cell suspension after dissociation. To obtain a large population of healthy dissociated cells, dissociation was stopped while a portion of whole animals were still undissociated. (F) A population of single cells generated by filtering dissociated sample. Scale bar is 5 μm. (G) Density plot shows clear separation by FACS of GFP positive and PI negative Z1/Z4 daughters, which are outlined by the green box.

Single-sex larval populations were harvested and cells dissociated at two time points during the first larval stage (L1) using a method we optimized as described in *Materials and Methods* (Figure 1). Because pure populations of XO larval males cannot be obtained, we generated pure populations of larval XX phenotypic males using a strain containing a conditional mutation in the sex determination gene *tra-2*. At the restrictive temperature this strain gives rise to XX pseudomale animals that are anatomically male and can sire progeny. We dissociated synchronized larvae and isolated either the somatic gonadal precursor cells (SGPs) Z1 and Z4 in mid-L1 or their daughters (Z1a/p and Z4a/p) toward the end of L1, after the Z1/Z4 divisions occurred in the vast majority of animals and when the gonad is overtly sexually dimorphic (Figure 2, C and D). In males, the first SGP division has a more pronounced asymmetry, giving rise to markedly smaller distal daughter cells (Z1a and Z4p). These distal daughters migrate posteriorly in the male, while the proximal daughter cells migrate anteriorly. In contrast, in the hermaphrodite there is no gonadal cell migration at this time. To enable isolation of the SGPs or their daughters by FACS, both strains also harbored an integrated array containing an *ehn-3a(promoter)*::*Venus* fusion, which is expressed exclusively in the Z1/Z4 lineage (Figure 2, A–D). These strains also contained a mutation in the *xol-1* gene, which kills spontaneous XO males and also enhances the phenotype of *tra-2* (Hodgkin 2002). We isolated 100–300 ng of RNA from 300,000–600,000 FACS-sorted gonadal cells per replicate (∼0.4 pg total RNA/cell).

### Optimization of RNA isolation

In initial experiments we isolated approximately 25 ng of total RNA from each sample. Because of the very small amount of RNA isolated, we first used Ovation RNA-Seq System V2 (NuGEN) to synthesize and amplify cDNA prior to RNA-seq. We found that the correlation of replicates was subpar using this method (Table 1). After optimizing our dissociation protocol (see *Materials and Methods*), we were able to harvest 100–300 ng of total RNA. We therefore tested whether RNA-seq using TruSeq (Illumina), which omits linear RNA amplification, might allow a better correlation of replicates. Indeed, TruSeq yielded high correlations of replicates even with a limited quantity of RNA (Table 1), and was therefore used for all experiments presented here.

**Table 1:** Correlation of replicates

Replicates Compared	Spearman’s Correlation, 9.5-hr Time Point	Spearman’s Correlation, 15-hr Time Point
Hermaphrodite gonadal cells	0.936, 0.939, and 0.966	0.962
Male gonadal cells	0.920, 0.930, and 0.935	0.929
Hermaphrodite whole animal	0.946, 0.909, and 0.912	0.963
Male whole animal	0.896, 0.913, and 0.946	0.968
Hermaphrodite gonadal cells, using NuGEN	0.877	

**Figure 3:**
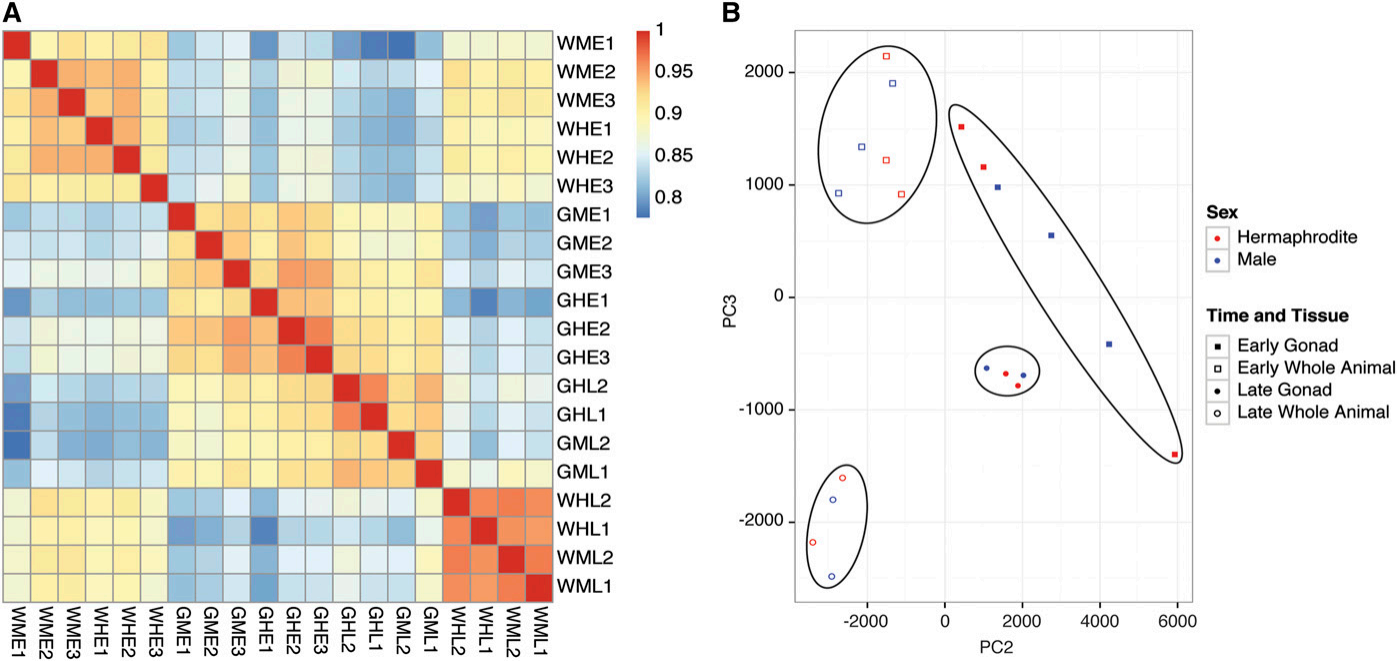
Replicates correlate highly and samples group by developmental time and tissue type but not sex. (A) Spearman’s correlation plot of the 20 samples in the study. Samples are clustered based on correlation. E, earlier time; G, gonadal cells; H, hermaphrodite; L, later time; M, male;  PC2, PCA Component 2;  PC3, PCA Component 3; W, whole animal. (B) Principal component analysis (PCA) two-dimensional scatter plot represents the RPKM values for each transcript in 20 individual samples. A scatter plot of the first and second principal component only separated samples by tissue and not time.

Samples were harvested in triplicate for the earlier time (profiling Z1/Z4) and in duplicate for the later time (profiling Z1/Z4 daughters). Spearman’s correlations of pairwise comparisons between the 20 samples confirmed a high correlation between replicates within the same sample (Figure 3A). At the earlier time there are few morphological differences between the sexes: males have a ventral coelomocyte located more posteriorly, they have four male-specific cephalic male (CEM) sensory neurons and they lack two hermaphrodite-specific neurons (HSNs) that undergo sex-specific programmed cell death during embryogenesis. Also the posterior B and Y blast cells are present in both sexes but have larger nuclei in males (Emmons 2005; Sulston and Horvitz 1977). Consistent with this limited anatomical dimorphism, fewer than 50 mRNAs exhibited sex-biased expression in whole larvae at the earlier time (Table 2). Our cutoff for enrichment was log_2_(twofold) with an uncorrected *P* value of < 0.025 and a false discovery rate (FDR) value < 0.05. In addition, while genetic experiments indicate that gonadal sex is determined prior to this time (Klass *et al.* 1976; Nelson *et al.* 1978), fewer than 30 transcripts showed sex-biased expression in SGPs at the earlier time point. At the later time we identified 269 sex-biased transcripts in the SGP daughters but only 27 in cells from whole larvae. This difference illustrates that sex-specific profiling of whole animals masks sex-biased expression limited to small numbers of cells.

**Table 2:** Number of transcripts that have sex-enriched or gonadally-biased expression

Differential expression	9.5-hr Time Point	15-hr Time Point
Hermaphrodite-enriched in gonadal cells	6	120
Male-enriched in gonadal cells	20	149
Hermaphrodite-enriched in whole animal	11	22
Male-enriched in whole animal	35	5
Gonadally-enriched in hermaphrodites	418	458
Gonadally-depleted in hermaphrodites	2047	2984
Gonadally-enriched in males	455	386
Gonadally-depleted in males	1908	2345

Principal component analysis (PCA) can indicate the degree of similarity between samples based on the degree of covariance. We performed a PCA of gene expression in the 20 samples based on the read depth for each gene [reads per kilobase of transcript per million mapped reads (RPKM)] and found that they grouped in four specific clusters. Developmental times and tissues were separable but samples from different sexes grouped together (Figure 3B). Sex differences likely were not separable by PCA because there were very few gene expression differences between the sexes at each time point for either whole body or gonadal cells (0.13%–1.4% of mRNAs had significant sex-biased expression). As a consequence, the high correlation of replicates (Figure S1) was critical for reliably identifying the rare sex-biased transcripts.

### Validation of SGP transcriptomes

To validate the gonadal transcriptome data (Table S2), we examined the representation of genes previously shown to be expressed in the SGPs. Among the mRNAs that were enriched in Z1/Z4 and their daughters relative to the entire larva, or expressed in Z1/Z4 and their daughters, to the best of our knowledge we identified all transcripts with previously described SGP expression. The majority of these transcripts were identified as significantly gonad-enriched in our analysis: *fkh-6* (Chang *et al.* 2004), *tra-1* (Mathies *et al.* 2004), *gem-4* (Church and Lambie 2003), *ceh-22* (Lam *et al.* 2006), *cyd-1* (Tilmann and Kimble 2005), *sys-1* (Miskowski *et al.* 2001), *dsh-2* (Chang *et al.* 2005), and *lin-26* (den Boer *et al.* 1998). Several other known SGP-expressed genes, [*gon-14* (Walker *et al.* 2004), *gon-2* (Sun and Lambie 1997), *gon-4* (Friedman *et al.* 2000), and *pop-1* (Siegfried *et al.* 2004)], also were identified as expressed in SGPs but did not meet our cutoff of log_2_(twofold) for enrichment in gonadal primordial cells due to their expression in other tissues. Based on our detection of previously described SGP-expressed genes, it appears that our data set identifies a majority of this gene class.

**Figure 4:**
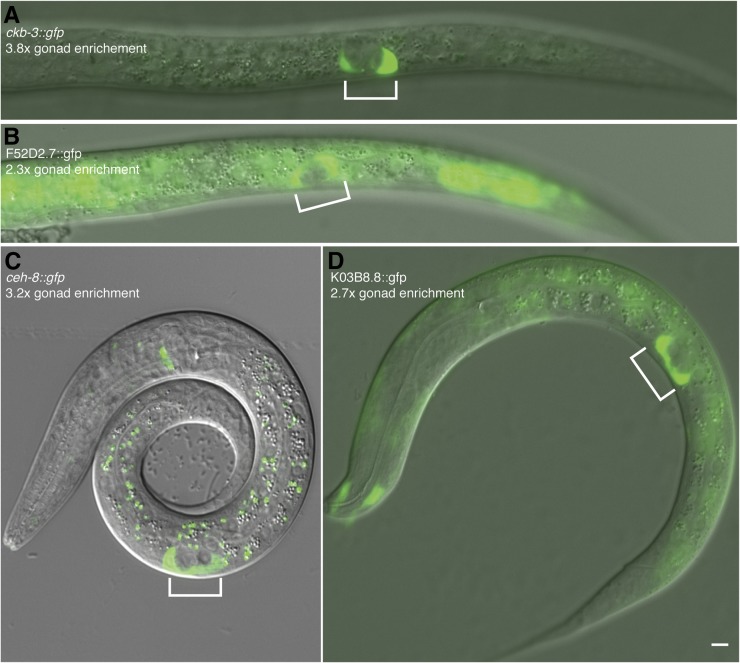
Transcriptional reporter gene analysis confirms gonadal mRNA enrichment. GFP expression of promoter fusions and log_2_(fold enrichment) values for Z1/Z4 compared to whole animal in hermaphrodites. (A) *ckb-3* reporter shows exclusive gonadal expression, (B) F52D2.7 reporter shows weak gonad-enrichment, (C) *ceh-8* reporter shows strong gonad-enrichment, and (D) K03B8.8 shows moderate gonad-enrichment. Gonads are indicated by brackets. Scale bar is 5 μm.

To further assess the SGP transcriptome data, we analyzed expression from transcriptional reporter transgenes containing from 400 bp to 3.6 kb of upstream sequences from each of ten genes showing SGP-enriched mRNA expression fused to GFP at the transcriptional start site. Seven of the ten reporters were expressed in the somatic gonadal precursor cells (*sdz-1*, R11A5.3, F52D2.7, *ckb-3*, K03B8.8, *ceh-8*, *pes-1*); the others (*pho-10*, *xtr-2*, T13F3.8) may have lacked essential regulatory elements, such as intronic enhancers, or might have been false positives. Among the reporters expressed in the gonad, some were expressed only in the Z1/Z4 lineage (Figure 4A), whereas others were expressed more broadly in other tissues (Figure 4B). In general, the specificity of reporter expression was reflected in the transcriptome analysis: the greater the fold of gonad-enrichment in our transcriptome analysis, the more exclusive the expression pattern of the reporter was to the gonad (Figure 4, compare panel A high enrichment *vs.* panel B lower enrichment).

Among the 269 sex-biased transcripts identified during the later time point, we detected all three of the previously described male-biased transcripts (*fkh-6*, *egl-5*, and *hlh-3*) as well as 146 novel male-biased transcripts (Table S3). Our transcriptome data recapitulated previous gonadal expression analyses. For example, an *fkh-6* reporter is initially expressed in Z1/Z4 in both sexes but after the Z1/Z4 division the reporter is maintained longer duration in male than hermaphrodite gonads (Chang *et al.* 2004). Likewise, transcriptome analysis found that *fkh-6* mRNA is gonad-enriched in both sexes at the earlier time, and at the later time it is gonad-enriched only in males. Additionally, reporters of *egl-5* and *hlh-3* were previously shown to be expressed in the late L1 gonad, exclusively in males (Kalis *et al.* 2010; J. Kimble, personal communication).

### Phenotypic analyses of male-biased, gonad-enriched transcripts

Of the mRNAs enriched in the gonad, 5.4% (earlier time) and 11.5% (later time) of the genes were previously reported to have a sterile phenotype based on either mutant alleles or RNAi. This relatively small proportion of gonadally-enriched mRNAs required for fertility may indicate significant genetic redundancy. In an attempt to uncover redundant requirements in gonadal function for the sex-biased and gonad-enriched transcripts expressed in the somatic gonad, we therefore performed RNAi of 44 male-biased, gonad-enriched mRNAs in four sensitized strains [(1) *tra-1(e1834)*; *fkh-6(ez16)*, (2) *him-8(e1489)*; (3) *egl-5(n486)*, *him-8(e1489)*; and (4) *hlh-3(ot354)*, *him-8(e1489)*] carrying mutations causing mild to more severe male gonadal defects. Three of the four genes are known to display partial redundancy with other gonadal regulators in males (Kalis *et al.* 2010). We tested more than 170 combinations of RNAi and sensitizing mutations without observing significant enhancement of gonadal defects (Table S4). To allow testing of many RNAi/strain combinations, we used an existing RNAi clone library (Kamath and Ahringer 2003) and administered RNAi by bacterial feeding. As a positive control for RNAi during each round of RNAi experiments we used *fkh-6(RNAi)*, which causes male-specific gonadogenesis defects in early larvae and spermathecal defects and infertility in adult hermaphrodites. The paucity of phenotypic effects in our RNAi assays further suggests extensive genetic redundancy within the gonad, and indicates that network-based approaches may be needed to elucidate the molecular functions of these genes.

### Identification of novel isoforms

**Figure 5:**
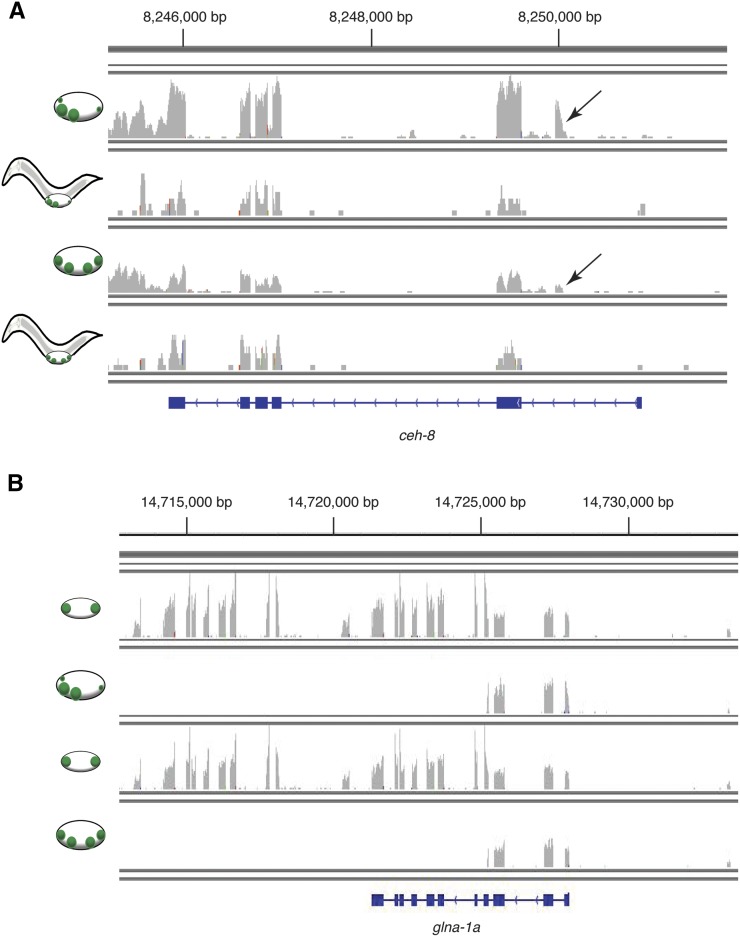
Identification of novel gonad-expressed exons. Plots of RNA-seq data; *x*-axis corresponds to the genomic position and *y*-axis denotes the average coverage of each position in the transcript normalized to the total number of uniquely mapped reads. (A) Normalized transcript coverage of the *ceh-8* locus in four samples: male Z1/Z4 daughter cells, male whole animals, hermaphrodite Z1/Z4 daughter cells, and hermaphrodite whole animals, respectively, from top to bottom. There was low expression for the *ceh-8* locus in whole animals so the *y*-axis values for whole animals were doubled to better visualize exon coverage. Arrow denotes novel gonad-specific exon. (B) Normalized transcript coverage of four samples of the *glna-1* genetic locus: male Z1/Z4 cells, male Z1/Z4 daughter cells, hermaphrodite Z1/Z4 cells, and hermaphrodite Z1/Z4 daughter cells.

Transcriptome analysis identified previously unannotated mRNA isoforms from 66 genes with gonad-enriched expression (20 unannotated isoforms were enriched at the earlier time, 14 at the later time, and 32 were enriched at both times, as indicated in Table S2 and Table S3). These unannotated isoforms used undescribed alternative transcriptional start sites or exons, (Figure 5A) or omitted specific exons that had previously appeared constitutive. Six mRNAs that were sex-biased in the SGP-daughters but not gonad-enriched were also expressed as unannotated isoforms. In contrast to a recent report of more than 300 sex-biased isoforms in the adult germline (Ortiz *et al.* 2014), we did not detect sex-biased mRNA isoforms in the somatic gonad; however, two genes (*glna-1* and *fbxc-40*), one of which is shown in Figure 5B expressed two distinct unannotated mRNA isoforms, one at the earlier time in Z1/Z4 and the other at the later time in Z1/Z4 daughters. It will be of interest to test whether these gonad-enriched isoforms have specialized functions.

### Correlations of transcripts with similar temporal, gonadal, or sex-biased expression

**Figure 6:**
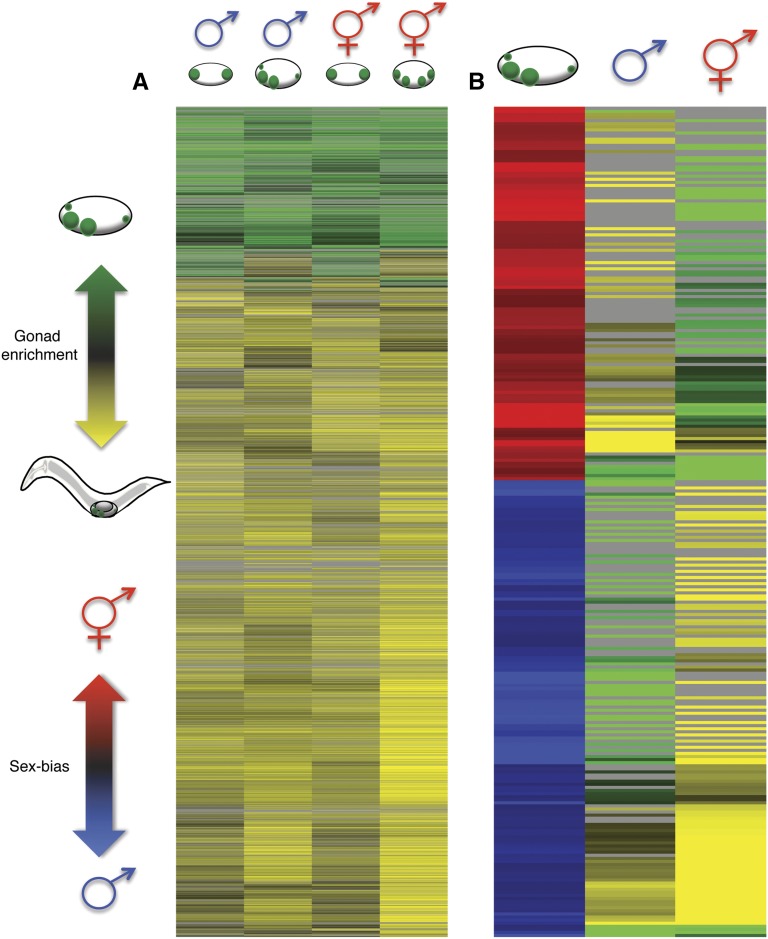
Gonadal-enrichment of transcripts becomes more diverse between the sexes with the division of Z1/Z4. (A) Heat map of 4689 mRNAs that were enriched or depleted in the gonad by at least log_2_(twofold) with *P* < 0.025 and false discovery rate (FDR) < 0.05 significance. Each row represents the enrichment of an individual transcript. Gonad enrichment is indicated by green and gonad depletion by yellow. Gray indicates that fold enrichment was not significant. Column 1: male gonadal-enrichment at the earlier time, column 2: male gonadal-enrichment later time, column 3: hermaphrodite gonadal-enrichment earlier time, and column 4: hermaphrodite gonadal-enrichment later time. (B) Heat map of 269 transcripts that were sex-biased within the gonad at the later time to at least log_2_(twofold) with *P* < 0.025 and FDR < 0.05 significance. Male-biased is indicated by blue and hermaphrodite-biased by red. Column 1: Sex-bias within the gonad at the later time, column 2: male gonad-enrichment, and column 3: hermaphrodite gonad-enrichment. Hierarchical cluster analysis was performed by Cluster 3.0 (Eisen *et al.* 1998) and visualized by Java TreeView (Saldanha 2004).

We next examined the pattern of transcript enrichment in the two sexes at each time. At the earlier time, gonadal transcriptomes were very similar in the two sexes, but they diverged at the later time (Table S2). Only 40% of mRNAs that were enriched in the gonad at the earlier time in either sex remained significantly enriched at the later time and many new mRNAs became gonad-enriched (Figure 6A, top portion of heatmap; Table S2), suggesting that the network of genes expressed during gonadal development is dynamic during this period. Of 269 mRNAs with sex-biased expression in the Z1/Z4 daughters, 29% also were enriched in the gonad and therefore may act primarily in that tissue (Table S3); 33% and 26% of mRNAs that were hermaphrodite-biased or male-biased, respectively, were enriched in the gonad, suggesting similar levels of sexual specialization in the gonads of each sex (Figure 6B).

### Candidate regulators of the gonadal transcriptome

We hypothesized that transcripts showing similar enrichment profiles might be regulated by the same *cis*-regulatory elements and *trans*-acting factors. Due to the generally compact nature of *C. elegans* promoter regions (Blazie *et al.* 2015), it is feasible to search for regulatory elements within undefined promoter regions. We defined the promoter region as 1000 bp upstream of the transcriptional start site and used DREME (Bailey 2011) to identify motifs overrepresented in the promoter regions of genes biased for expression in one sex and underrepresented in the promoter regions of genes biased for expression in the other sex. The promoter regions of hermaphrodite-biased genes expressed in Z1/Z4 daughter cells were enriched for the motif ACTTKAAC, present in 18% of 120 hermaphrodite-biased genes (Table S3), while this motif appeared in less than 1% of the promoter regions of male-biased genes at the same time point. We then used FIMO (Grant *et al.* 2011) to identify the promoter regions of hermaphrodite-enriched genes that contained the ACTTKAAC motif (indicated in Table S3). Further analysis using Tomtom (Gupta *et al.* 2007) to compare the hermaphrodite-enriched motif ACTTKAAC against a database of known motifs identified HLH-2 as a potential *trans*-acting factor binding this motif. We found *hlh-2* mRNA to be expressed in the somatic gonad in both sexes. However, HLH-2 has been shown to act as a DNA-binding heterodimer (Portman and Emmons 2000; Thellmann *et al.* 2003), indicating that HLH-2 could be acting through the hermaphrodite-enriched motif, presumably in concert with other, sex-limited, factors. Of the seven genes with which HLH-2 is known to interact (*ces-1*, *egl-43*, *hlh-3*, *hlh-14*, *lag-2*, *lin-12*, *lin-32*) only one gene, *hlh-3*, has sex-limited expression in the somatic gonadal precursor cells, with higher expression in male cells. This could indicate that an HLH-3/HLH-2 heterodimer acts in males to repress hermaphrodite-biased transcripts or alternatively HLH-2 may have another partner, yet to be identified, in the gonad.

We did not identify an enriched motif in male-biased transcripts but previous work has identified two transcriptional regulators, TRA-1 and FKH-6, which are crucial for male-specific gonadal development. Some of the male-biased transcripts identified here are likely to be regulated in the somatic gonad by one or both of these transcription factors. TRA-1 (Zarkower and Hodgkin 1992) is a GLI homolog that regulates sex determination by repressing genes involved in male differentiation (Chen and Ellis 2000; Conradt and Horvitz 1999; Yi *et al.* 2000). TRA-1 is required only in hermaphrodites in most tissues, but in the somatic gonad TRA-1 not only promotes hermaphrodite development in XX animals but also acts in both sexes to regulate SGP position and polarity and promote proliferation of gonadal cells (Mathies *et al.* 2004; Schedl *et al.* 1989). To identify potential mediators of TRA-1-dependent gonadal development, we asked whether any of the sex-biased gonadal transcripts identified here also are likely direct targets of TRA-1. A recent study identified 184 *in vivo* TRA-1 binding sites in XX larvae (Berkseth *et al.* 2013). Four of the 149 male-biased gonadal transcripts (*ztf-22*, *cec-1*, *col-176*, and C26B9.6) were from genes near TRA-1 binding sites, which was a statistically significant hyper-geometric probability of overlap between these two data sets. Additional mRNAs that were enriched in the gonad and near TRA-1 binding sites are noted in Table S2. Future experiments can test whether these genes are downstream effectors of TRA-1 in the SGPs. Because the previous TRA-1 ChIP-seq was performed on L2 and later larvae there may be additional TRA-1 SGP targets during L1 that were not identified. FKH-6 also is essential for proper differentiation of somatic cells in the male gonad and functions in parallel with TRA-1 to promote cell proliferation in the male somatic gonad (Chang *et al.* 2004). We investigated the occurrence of FKH-6 binding sites in genes with gonad-enriched expression, but the low sequence complexity of the FKH-6 binding site (Kalis *et al.* 2010) precluded useful enrichment analysis. Determining the direct targets of gonadal transcription factors may provide valuable entry points to identify the regulatory networks controlling sex-specific gonadal gene expression during this period.

**Figure 7:**
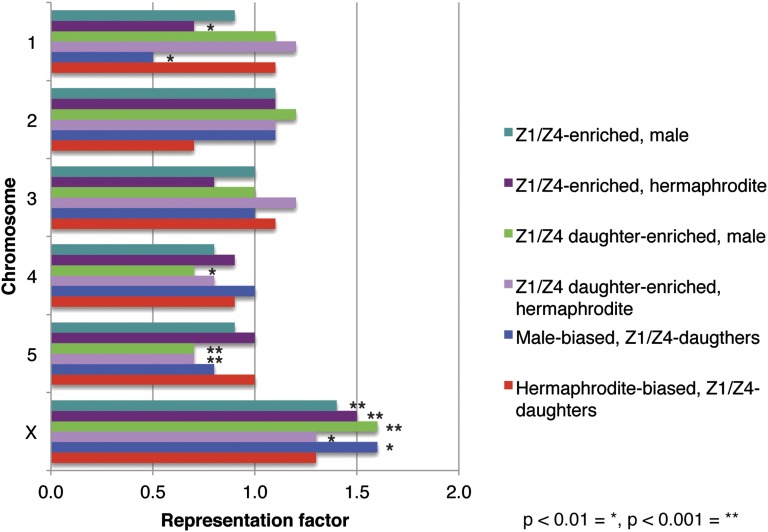
Gonad-enriched transcripts were significantly enriched on the X chromosome. Chromosomal distribution of gonad-enriched transcripts in either Z1/Z4 or Z1/Z4-daughters (for either the male or hermaphrodite) and sex-biased transcripts. The representation factor is the ratio of observed to expected transcripts for each chromosome. A hyper-geometric probability test was used to determine any statistically significant deviation of observed from expected. *P* values of < 0.01 and 0.001 are indicated by * and **, respectively.

Previous studies found that genes with female-biased expression in the adult soma are enriched on the X chromosome in adult animals, while no bias was observed in adult males or larval animals of either sex (Reinke *et al.* 2004; Thoemke *et al.* 2005). We therefore examined chromosomal locations of the sex-biased and gonad-enriched transcripts identified in this study. We found that gonad-enriched genes from both sexes and both time points were significantly enriched for expression on the X chromosome. Additionally transcripts that were male-biased in Z1/Z4 daughters were also significantly enriched on the X chromosome (Figure 7).

### Enrichment of functional gene classes

Gene ontology analysis of mRNAs enriched in Z1/Z4 and their daughters revealed the anticipated enrichment for GO terms related to reproductive processes (2.18- and 3.04-fold enrichment compared to whole animal for Z1/Z4 and their daughters, respectively; for mapped genes, see *Materials and Methods*), gonad development (2.33- and 3.81-fold enrichment) and sex differentiation (2.31- and greater than 5-fold enrichment). We also found more specialized functions, some of which were enriched in only one sex. In particular, the male-biased gonadal transcriptome was enriched for expression of genes involved in cell migration, consistent with the male-specific cell movements that occur during this period (Table S3). Additionally, the male-biased gonadal transcriptome included a number of kinases, *Patched*-related genes and homeobox genes, which may provide insights into mechanisms of male-specific gonadal development. Finally, the hermaphrodite-biased gonadal transcriptome was enriched for expression of endopeptidase inhibitors while the male-biased transcriptome was enriched for expression of peptidases, suggesting a possible post-translational mechanism for gonadal sex differentiation.

### Sex-biased gonadal expression shared in worm and mouse

Gene expression at the onset of gonad sexual differentiation also has been studied in the mouse (Jameson *et al.* 2012). To ask whether related genes may function in gonadal differentiation in nematodes and mammals, we compared sex-biased gonadal transcripts from the two species. Approximately 10% of the *C. elegans* male-biased transcripts identified in this study have mammalian orthologs with male-biased expression in somatic supporting cells of the early fetal gonad of the mouse, while 3% of the *C. elegans* hermaphrodite-biased transcripts have mouse orthologs with female-biased expression in supporting cells (Table S3).

## Discussion

We have described an optimized method for single cell transcriptional profiling of *C. elegans* larvae that we have used to define the transcriptomes of the SGPs and their daughters in each sex, identifying many mRNAs whose expression is enriched in the early SGP lineage in one or both sexes. Transcriptome analysis identified all of the genes previously known to be specifically expressed in Z1/Z4 and their daughters as well as all of the genes previously known to be essential for early larval gonadal development, suggesting that the transcriptomes we generated include the majority of transcripts strongly enriched in the early larval somatic gonad. We also identified a number of previously unannotated mRNA isoforms. The times analyzed include the onset of sex-specific gonadal development and comparison of Z1/Z4 transcriptomes with those of their daughters less than 6 hr later revealed the emergence of a suite of sex-biased transcripts that are likely to mediate differential gonadal development in the two sexes. The sex-biased expression of homologous genes in somatic supporting cells of the gonad in both *C. elegans* and mice, particularly in males, may indicate evolutionary conservation of part of the somatic gonad sexual differentiation program.

Profiling isolated cells identified ten times more sex-biased transcripts than whole animal analysis during the same period. Due to this higher sensitivity, cell type specific transcriptome analysis is likely to have broad utility in elucidating developmental programs in other tissues and organs. In principle, cell type specific enrichment and sex bias can be determined for any larval cell type where appropriate sorting markers are available. Attractive targets for such analysis would be larval blast cells with highly dimorphic development, such as the M, T, or B cells (Sulston and Horvitz 1977). Cell type specific transcriptome analysis can also allow much more sensitive analysis of the effects of mutations or environmental factors in cell types of particular interest. For example, mutational effects have previously been studied by transcriptome comparisons in the VA neural cells (Von Stetina *et al.* 2007).

We performed extensive RNAi and mutant analysis (data not shown) of the gonad-enriched genes, focusing on those with male-biased expression. Strikingly, RNAi of male-biased mRNAs revealed almost no new phenotypes even when RNAi was performed on sensitized backgrounds. In light of the strong Z1/Z4 lineage enrichment of the transcripts tested, this result suggests a high level of functional redundancy in the early somatic gonad and/or minor roles in gonadal development for many gonad-enriched mRNAs. Given the direct role of the gonad in reproduction, a genetic buffering system to prevent severe effects from the loss of many gonadally-expressed genes would perhaps be expected. Future studies may need to employ network-based approaches to elucidate the functional roles of the gonad-enriched transcripts we have identified. Possible approaches include focusing on functional classes (*e.g.*, Table S3) or gonadal gene regulatory networks. As an entry point to determine potential genetic networks, transcripts with similar expression profiles may be useful. Transcripts that have similar patterns for gonadal enrichment and sex-biased expression could be regulated by common factors and therefore contain similar regulatory elements. One example of transcripts that could be regulated in the same manner is the group of four at the bottom of the heat map in Figure 6B. These transcripts are male-biased, yet they are gonad-enriched compared to the whole animal in both males and hermaphrodites and could be regulated by a similar mechanism. Identifying the *cis*-regulatory elements and *trans*-acting regulators, which should be expressed in the gonad, could better define the shape of the gonadal genetic networks. The enrichment of a putative HLH-2 binding site in promoters of hermaphrodite-biased genes may provide a first step. Analyzing the direct targets of key transcriptional regulators including HLH-2, TRA-1, and FKH-6 may provide insight into how the gonadal sex differences are established.

We identified more than 70 genes that have isoforms that were not previously annotated. In many cases the annotated isoform is expressed in the whole animal while the gonadal cells express a novel isoform (Figure 5A). It will be important to determine whether these novel isoforms provide gene functions specific to gonadogenesis. While the *C. elegans* genome has been well studied it is likely that transcriptome analysis in other cell types also will identify additional novel mRNA isoforms with restricted expression domains.

### Conclusion

To our knowledge these data represent the only sex-specific transcriptome analysis of a *C. elegans* somatic tissue. We identified several hundred gonad-enriched transcripts, including a number of novel isoforms, as well as a small suite of sex-biased transcripts. The transcriptomes we report provide insights into the processes that drive early gonadal development and should provide a useful resource to further define how development of the bipotential *C. elegans* SGPs is coordinated to form two distinct organs with very different organizations and functions.

## 

## Supplementary Material

Supporting Information
