# Supplementary material for: Cell-Specific mRNA Profiling of the Caenorhabditis elegans Somatic Gonadal Precursor Cells Identifies Suites of Sex-Biased and Gonad-Enriched Transcripts
Source: G3 (Bethesda). 2015 Oct 23;5(12):2831–41. doi: 10.1534/g3.115.022517 (PMC4683654; doi:10.1534/g3.115.022517)
Supplement: Supporting Information [file supp_5_12_2831__index.html]

Cell-Specific mRNA Profiling of the Caenorhabditis elegans Somatic Gonadal Precursor Cells Identifies Suites of Sex-Biased and Gonad-Enriched Transcripts — Supporting Information 

# Cell-Specific mRNA Profiling of the *Caenorhabditis elegans* Somatic Gonadal Precursor Cells Identifies Suites of Sex-Biased and Gonad-Enriched Transcripts

## Supporting Information for Kroetz and Zarkower *et al.*, 2015

**Files in this Data Supplement:**

- Figure S1 - RNA-seq from replicates of SGP cells are concordant. (.tif, 1,378 KB)
- Table S1 - Primer sequences used for transcriptional reporters. (.xlsx, 49 KB)
- Table S2 - Gonad-enriched transcripts. Enrichment for each developmental time and sex are on separate tabs. Negative fold change values indicate male-bias and gonadenrichment.Positive fold change values indicate hermaphrodite-bias and gonaddepletion. Greyed boxes indicate fold changes with a FDR > 0.05 (not significant.). (.xlsx, 294 KB)
- Table S3 - Sex-biased transcripts in gonadal cells at later time point. Negative fold change values indicate male-bias and gonad-enrichment. Positive fold change values indicate hermaphrodite-bias and gonad-depletion. Greyed boxes indicate fold changes with a FDR > 0.05 (not significant.). (.xlsx, 100 KB)
- Table S4 - Genes tested by RNAi for gonadal defects. RNAi was conducted in four different sensitized backgrounds [(1) *tra-1(e1834)*; (2) *fkh-6(ez16)*, *him-8(e1489)*; (3)  *egl-5(n486)*, *him-8(e1489)*; and (4) *hlh-3(ot354)*, *him-8(e1489)*] to determine whether there was enhancement of the male gonadal defects of each strain. (.xlsx, 36 KB)
