# Supplementary figures and images for: Cell-Specific mRNA Profiling of the Caenorhabditis elegans Somatic Gonadal Precursor Cells Identifies Suites of Sex-Biased and Gonad-Enriched Transcripts
Source: G3 (Bethesda). 2015 Oct 23;5(12):2831–41. doi: 10.1534/g3.115.022517 (PMC4683654; doi:10.1534/g3.115.022517)

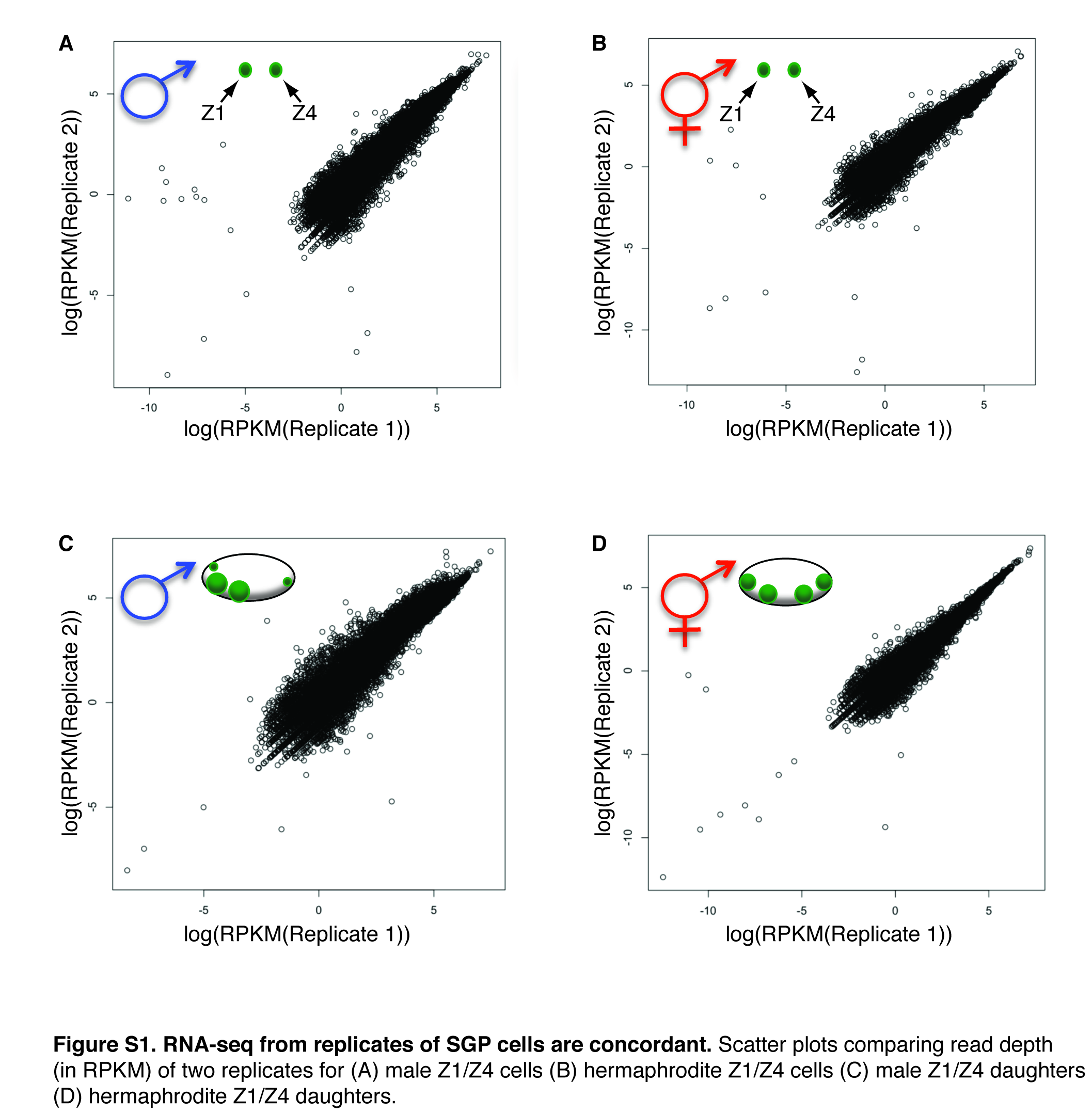

Supplement: Supporting Information [file supp_g3.115.022517_FigureS1.tif]
